# Circulating CD40 and sCD40L Predict Changes in Renal Function in Subjects with Chronic Kidney Disease

**DOI:** 10.1038/s41598-017-08426-8

**Published:** 2017-08-11

**Authors:** Jeffrey X. Xie, Helen Alderson, James Ritchie, Philip A. Kalra, Yanmei Xie, Kaili Ren, Hanh Nguyen, Tian Chen, Pamela Brewster, Rajesh Gupta, Lance D. Dworkin, Deepak Malhotra, Christopher J. Cooper, Jiang Tian, Steven T. Haller

**Affiliations:** 1 University of Toledo College of Medicine and Life Sciences, Toledo, OH, United States; 2 Salford Royal Hospital, Salford, United Kingdom

## Abstract

Soluble CD40 ligand (sCD40L) has been implicated in the development of renal injury. The CD40 receptor exists in a soluble form, sCD40R, and has been shown to function as a competitive antagonist against CD40 activation. We analyzed whether plasma levels of sCD40L and sCD40R predict changes in renal function in an all-cause chronic kidney disease (CKD) cohort. Stratification of subjects based on sCD40L and sCD40R individually, as well as in combination, demonstrated that sCD40L was directly associated with declines in estimated glomerular filtration rate (eGFR). sCD40R was negatively associated with declines in eGFR. Baseline characteristics following stratification, including systolic blood pressure, history of diabetes mellitus or peripheral vascular disease, primary renal disease classification, and angiotensin converting enzyme inhibitor or angiotensin receptor blocker usage were not significantly different. High sCD40L and low sCD40R were both found to be independent predictors of a decline in eGFR at 1-year follow-up (−7.57%, p = 0.014; −6.39%, p = 0.044). Our data suggest that circulating levels of sCD40L and sCD40R are associated with changes in renal function in patients with CKD. The CD40 decoy receptor, sCD40R, may serve as a potential therapeutic target to attenuate renal function decline.

## Introduction

Chronic Kidney Disease (CKD) is defined as a glomerular filtration rate (GFR) below 60 mL/min per 1.73 m^2^ for more than three months^1^. A recent study estimated the prevalence of CKD in the United States at over 13%^2^. Several molecular pathways have been investigated for their involvement in the development of CKD, but the exact mechanisms that drive CKD progression remain unknown.

CD40 was originally discovered to mediate humoral immune interactions^3^. However, it is now established that CD40 is expressed by a multitude of cell types including renal proximal tubule epithelial cells and is known to mediate pro-inflammatory and pro-fibrotic pathways^4–7^. The soluble ligand of the CD40 receptor, sCD40L, is largely derived from activated platelets^8, 9^. The CD40 receptor can also exist in a soluble form, sCD40R, and has been shown to function as a competitive antagonist, or decoy receptor, to CD40^10–13^.

CD40 signaling events have been linked to atherosclerosis and thrombosis, processes which are thought to develop in part due to persistent inflammation^14–18^. Interestingly, we have shown that subjects with atherosclerotic renal artery stenosis have significantly elevated levels of circulating sCD40L^19^. Moreover, we have shown that higher levels of baseline circulating sCD40R were associated with a higher 1-year follow-up estimated GFR (eGFR), presumably due to the ability of sCD40R to antagonize CD40-mediated signaling^20^. As atherosclerotic renal artery stenosis is a known cause of CKD^21^, the current study builds upon our previous findings by broadening the scope of our study from renal artery stenosis to an all-cause CKD population.

The primary aim of the present study is to investigate whether circulating levels of sCD40L and sCD40R are associated with changes in renal function in subjects with CKD. As a secondary aim, we analyzed the effects of plasma levels of sCD40L and sCD40R on renal and cardiac mortality, rate of renal replacement therapy (RRT), myocardial infarction, stroke, and heart failure incidence.

## Subjects and Methods

### Patient Population

1750 patients were recruited to the Chronic Renal Insufficiency Standards and Implementation Study (CRISIS) prior to January 31^st^, 2010, with a median follow up time of 4.5 years (IQR 2.9–6.9). Baseline plasma samples were obtained from 250 randomly selected patients from this group at the time of their enrollment into CRISIS. CRISIS is a prospective observational study of outcomes in an all-cause CKD population from Salford, Greater Manchester, UK. This study was carried out in accordance with all relevant guidelines and regulations as approved by the National Health Service Research and Ethics Committee and the University of Toledo Institutional Review Board. All participants provided written informed consent. Patients referred to the nephrology clinic aged 18 years and older with a GFR <60 mL/min/1.7 m^2^ and without immediate need for dialysis were considered for the study. Glomerular filtration rate (eGFR) was estimated using the Modification of Diet in Renal Disease Study (MDRD) equation and the serum creatinine Chronic Kidney Disease Epidemiology Collaboration (CKD-EPI) equation. MDRD eGFR is reported unless stated otherwise. Full methodology for CRISIS has been previously detailed^22–24^.

### Measurement of sCD40L and sCD40

Baseline plasma levels of sCD40L and sCD40R were measured by enzyme-linked immunosorbent assay (Abcam Inc, Cambridge, MA [CD40]; and R&D Systems, Minneapolis, MN [sCD40L]) at the University of Toledo according to the manufacturer recommendations. Full methodology has been described previously^20^.

### Study Aims

The primary aim of our study was to analyze whether plasma levels of sCD40L and sCD40R were associated with longitudinal changes in renal function over 4 years of follow-up. As a secondary analysis, we determined the effects of plasma levels of sCD40L and sCD40R on the composite endpoint, defined as the first occurrence of renal replacement therapy (RRT), cardiovascular or renal death, or major cardiovascular adverse events (MACE) consisting of either myocardial infarction, congestive heart failure or stroke. Full methodologies for the validation of renal function measurements and definition of composite endpoint events have been described previously^22–24^.

### Statistical Analysis

Assuming a minimum detectable correlation coefficient of r = 0.17 between high levels of sCD40L and low levels of sCD40R with decline in percent change in eGFR from baseline, the sample size needed was 250 to achieve 80% power at one-sided 5% significance level allowing for 15% attrition.

Continuous variables are presented as either mean ± standard deviation or medians with interquartile range (IQR), while categorical variables are presented as frequency and percent for each level. Pearson correlation coefficient was used to evaluate the relationship between baseline sCD40L and sCD40R. Comparisons of grouped continuous data were performed using ANOVA with post hoc pairwise comparison using Tukey-Kramer multiple comparisons test, and two-sample *t*-tests. For categorical variables, the Fisher exact test was used due to low frequency of counts for some factors.

Subjects were dichotomized based on plasma levels of either sCD40L or sCD40R into high (greater than median) or low (less than or equal to median) groups, and were used to compare average percent change in eGFR from baseline to 1-year follow-up. Subjects were stratified by the combination of both median sCD40L and sCD40R, in order to evaluate the combined effect of sCD40L and sCD40R on CKD progression. Further stratification, such as the use of quartiles, was deemed a less favorable approach due to our sample size and the complexity of the model with a total of 16 combinations when combined sCD40L and sCD40R was evaluated. Multivariable linear regression adjusted for age, sex, baseline eGFR, systolic blood pressure (SBP), peripheral vascular disease (PVD), primary renal disease classification, and use of ACEi/ARB or aspirin medication at baseline was employed to examine the effect of sCD40L and sCD40R as dichotomous predictors of the percent change in renal function from baseline to 1-year follow-up. Backward stepwise methodology was used to select factors for the model. All multivariable models were tested for interaction between predictors and were found to be non-significant. Odds ratios and corresponding 95% confidence intervals for each predictor in the multivariable linear regression models on continuous outcomes were calculated following the method proposed by Moser *et al*.^25^.

Time-to-event outcomes, including the composite and secondary study endpoints, were examined using Kaplan-Meier estimates with the log-rank statistic used to compare the high and low medians for both the sCD40L and sCD40R groups. Hazard risk ratios with 95% confidence intervals were examined using the Cox proportional-hazards model for both groups adjusted for age, sex, body mass index (BMI), and baseline creatinine and urine protein. Event rates and associated odds ratios were compared between the high and low groups for both sCD40L and sCD40R.

The longitudinal effect of dichotomized sCD40L and sCD40R on percent change in renal function from baseline to annual follow-up out to 4 years was assessed using generalized estimating equation (GEE) analysis. The GEE model was employed to address the dependency among repeated measurements of renal function from the same subject. We opted to use GEE analysis, rather than a parametric alternative such as the generalized linear mixed-effects models (GLMM), because GEE estimates are robust for a wider class of data distributions^26^. The model was adjusted for the following factors: age, sex, SBP, baseline GFR, history of PVD and diabetes mellitus, primary renal disease classification, and use of angiotensin converting enzyme inhibitor (ACEi), angiotensin receptor blocker (ARB) or aspirin. Because of missing observations due to censoring by drop-outs, missed visits or death, weighted GEE (WGEE) was applied to adjust the analysis using the inverse probability-weighted method to account for missing observations.

All analyses were performed in SAS 9.4 and R 3.0. Statistical significance was defined as a p-value < 0.05.

## Results

### Study Population

Baseline levels of the study population are presented in Table 1. For analysis of this population, eGFR, sCD40L, and sCD40R were available in 243 out of 250 total subjects. Due to extreme observations in plasma levels of sCD40L and sCD40R, natural logarithmic transformation did not achieve sufficient normality. Receiver operating characteristic (ROC) analyses did not yield a significant predictor cut point with an acceptable (>60%) area under the curve (AUC). Therefore, to reduce the effect of outliers, subjects were stratified into two groups based on their plasma levels of sCD40L: high (greater than median) and low (less than or equal to median) (>205.0 [142.4, 324.6] pg/mL and ≤ 205.0 [142.4, 324.6] pg/mL respectively). We have previously reported that the average plasma concentration of sCD40L in a cohort of 30 normal controls was 65.2 ± 7.4 pg/mL^19^. The high and low sCD40L groups were well matched at baseline. There were no statistically significant differences in levels of sCD40R, age, sex, body mass index (BMI), or medication use. Importantly, the primary cause of CKD did not differ between the high and low sCD40L groups. However, a statistically significant difference in diastolic blood pressure (DBP) was detected between these two groups (74.8 ± 12.7 mmHg and 71.7 ± 11.3 mmHg; p = 0.04). Median values for sCD40L and sCD40R were not significantly different among the types of primary renal disease (S Table 1).

**Table 1:** Baseline Clinical Characteristics among Participants in the CRISIS Clinical Trial.

Characteristics^*^	High sCD40L (>205.0 [142.4, 324.6] pg/mL) (*n* = 122)	Low sCD40L (≤205.0 [142.4, 324.6] pg/mL) (*n* = 121)	P-value	High sCD40R (>81.4 [50.9, 306.0] pg/mL) (*n* = 122)	Low sCD40R (≤81.4 [50.9, 306.0] pg/mL) (*n* = 121)	P-value
Age (yr)	65.84 ± 14.99	66.05 ± 13.54	0.91	63.80 ± 15.69	68.10 ± 12.34	0.02
Male	74 (61%)	71 (59%)	0.79	76 (62%)	69 (57%)	0.48
Body mass index (kg/m^2^)	27.38 ± 5.68	28.41 ± 5.11	0.17	27.37 ± 4.57	28.53 ± 6.14	0.13
Smoking (current)	15 (12%)	12 (10%)	0.56	13 (11%)	14 (12%)	0.82
Systolic BP (mmHg)	136.76 ± 20.87	136.87 ± 20.18	0.97	134.8 ± 20.61	138.8 ± 20.25	0.12
Diastolic BP (mmHg)	74.79 ± 12.66	71.74 ± 11.26	0.04	73.93 ± 13.42	72.60 ± 10.53	0.39
MDRD eGFR (ml/min per 1.73 m^2^)	28.76 ± 14.70	29.30 ± 15.19	0.78	29.30 ± 14.62	30.02 ± 15.63	0.71
CKD-EPI eGFR (ml/min per 1.73 m^2^)	31.56 ± 17.67	32.02 ± 18.1	0.84	31.34 ± 17.4	32.24 ± 18.4	0.69
Urine protein (mg/dL)	60.78 ± 111.23	54.09 ± 105.96	0.63	61.96 ± 124.5	52.90 ± 89.79	0.52
Creatinine (mg/dL)	2.83 ± 1.68	2.74 ± 1.60	0.68	2.82 ± 1.61	2.75 ± 1.67	0.75
History of Myocardial infarction	18 (15%)	23 (19%)	0.40	23 (19%)	18 (15%)	0.51
History of Angina	23 (19%)	25 (21%)	0.87	25 (20%)	23 (19%)	0.92
History of CVA	12 (10%)	6 (5%)	0.22	6 (5%)	12 (10%)	0.21
History of TIA	11 (9%)	10 (8%)	>0.99	7 (6%)	14 (12%)	0.16
History of Diabetes mellitus	37 (30%)	42 (35%)	0.50	33 (27%)	46 (38%)	0.09
History of Peripheral vascular disease	22 (18%)	23 (19%)	0.87	20 (16%)	25 (21%)	0.49
ACEi	43 (35%)	52 (43%)	0.24	51 (42%)	44 (36%)	0.46
ARB	31 (25%)	30 (25%)	>0.99	24 (20%)	37 (31%)	0.07
ACEi or ARB	77 (64%)	71 (58%)	0.43	74 (61%)	74 (61%)	>0.99
β-Blocker	36 (30%)	36 (30%)	>0.99	38 (31%)	34 (28%)	0.70
Diuretic	53 (43%)	59 (49%)	0.44	61 (50%)	51 (42%)	0.27
Statin	68 (56%)	72 (60%)	0.60	63 (52%)	77 (64%)	0.08
Aspirin	55 (45%)	48 (40%)	0.44	37 (30%)	66 (55%)	<0.001
Mean sCD40L (pg/mL)	479 ± 644	137 ± 37	<0.001	357 ± 652	261 ± 212	0.12
Mean sCD40R (pg/mL)	662 ± 1940	596 ± 2047	0.80	1200 ± 2695	53 ± 13	<0.001
Mean log sCD40L	5.93 ± 0.56	4.88 ± 0.30	<0.001	5.46 ± 0.76	5.35 ± 0.61	0.21
Mean log sCD40R	5.04 ± 1.41	4.82 ± 1.34	0.22	5.91 ± 1.34	3.94 ± 0.24	<0.001
**Primary renal disease**						
DM	22 (18%)	18 (15%)	0.70	16 (13%)	24 (20%)	0.59
APKD	5 (4%)	11 (9%)	0.29	10 (8%)	6 (5%)	0.39
GN/VAS	14 (11%)	18 (15%)	0.74	18 (15%)	14 (12%)	0.50
Pyelonephritis	12 (10%)	4 (3%)	0.11	10 (8%)	6 (5%)	0.39
VAS/HTN	41 (34%)	42 (35%)		39 (32%)	44 (36%)	
Other	28 (23%)	28 (23%)	>0.99	29 (24%)	27 (22%)	0.70

Subjects are stratified by median plasma levels of sCD40L or sCD40R.

*Data are expressed as the mean ± SD or number (percentage). Comparisons were evaluated using two sample t-test for continuous data and Fisher’s exact test for categorical data.

*Abbreviations*: *sCD40L, soluble CD40 ligand; sCD40R, soluble CD40 receptor; yr, year; m, meter; kg, kilogram; BP, blood pressure; MDRD, Modification of Diet in Renal Disease; CKD-EPI, Chronic Kidney Disease Epidemiology Collaboration; eGFR, estimated glomerular filtration rate; CVD, cerebral vascular accident; TIA, transient ischemic accident; ACEi, angiotensin converting enzyme inhibitor; ARB, angiotensin receptor blocker; and β-Blocker, beta adrenergic blocker*

*DM, diabetic glomerularnephritis; APKD, adult polycystic kidney disease; GN/VAS, glomerularnephritis vasculitis; VAS/HTN, vascular hypertension.*

We also assessed the effects of plasma levels of the CD40 decoy receptor, sCD40R, on kidney function in this population. In order to conduct these analyses, subjects were dichotomized by either high or low sCD40R (>81.4 [50.9, 306.0] pg/mL and ≤ 81.4 [50.9, 306.0] pg/mL respectively). It has been reported that in a cohort of 205 control subjects, the average plasma sCD40R concentration was 41.7 ± 13.2 pg/mL^27^. The high and low sCD40R groups were well matched at baseline (Table 1), with the exception of age (63.8 ± 15.7 years and 68.1 ± 12.3 years; p = 0.02) and aspirin use (30% and 55%; p < 0.001).

### CD40 and Renal Function

**Figure 1:**
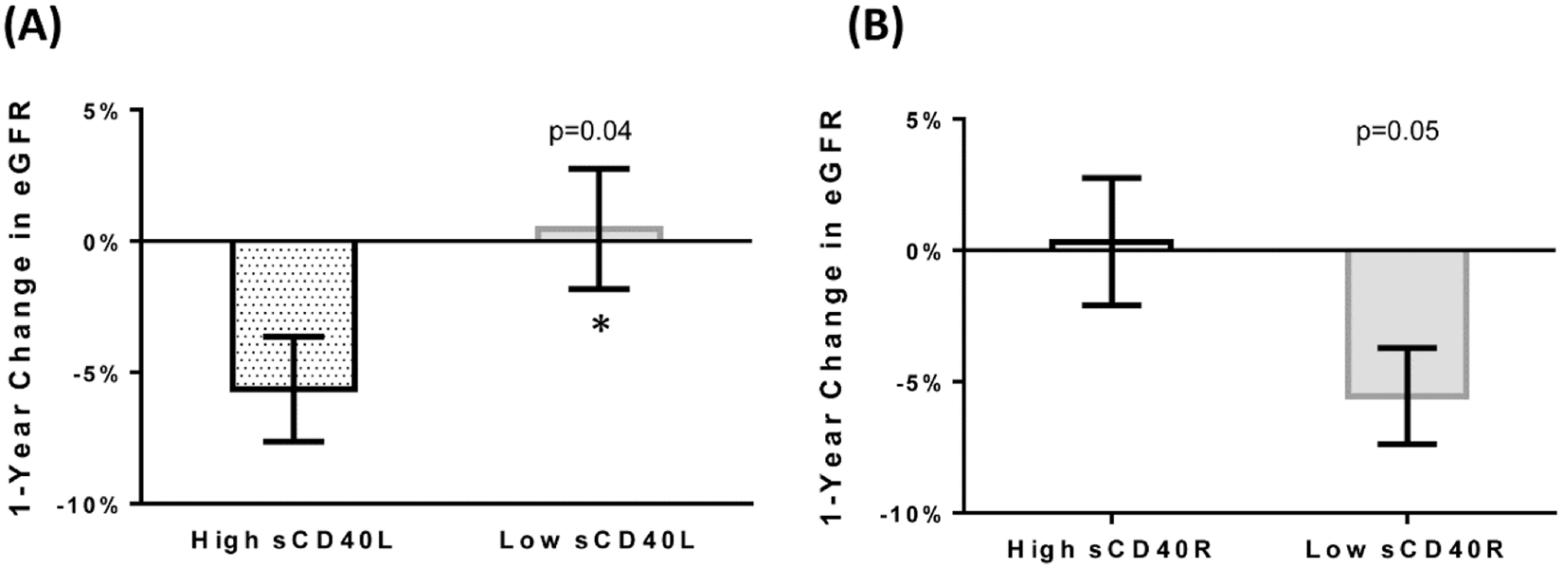
Percent Change in eGFR in the CRISIS Cohort Dichotomized by Median sCD40L or sCD40R at 1-Year Follow-up. (**A**) Subjects were dichotomized by median sCD40L (205.0 [142.4, 324.6] pg/mL) or (**B**) by median sCD40R (81.4 [50.9, 306.0] pg/mL).

**Figure 2:**
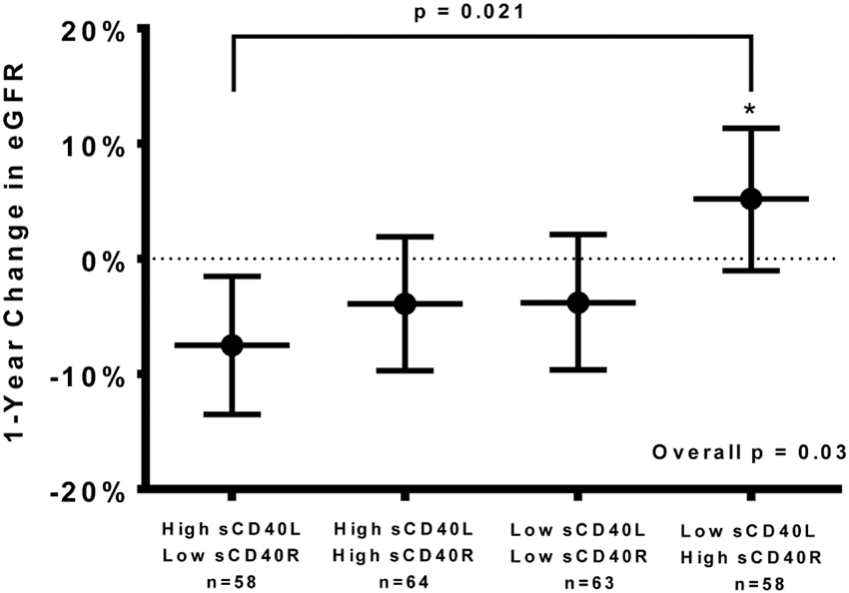
Comparison of 1-year percent change in eGFR between the combined sCD40L and sCD40R groups. Subjects were stratified into four groups based on median plasma levels of sCD40L and sCD40R. Data is presented as the mean ± 95% confidence interval.

Ninety-seven percent of the study cohort had moderate to end-stage-renal-disease (stages III 42%, IV 34% or V 21%). The average baseline eGFR was 32 ± 18 ml/min per 1.73 m^2^ and the average percent decline in eGFR at 1-year follow-up was −3.4% ± 23.5%. Only 10% had a 1-year decline of 30% or more. Subjects with high sCD40L had a greater percent decline in eGFR at 1-year follow-up compared with subjects with low sCD40L (−5.6% ± 0.21% vs 0.47% ± 0.23%, p = 0.04) (Fig. 1a). Since sCD40R is hypothesized to function as a circulating decoy receptor, we assessed the relationship between this biomarker and change in renal function at 1-year follow-up. There was a trend towards subjects with low sCD40R having a greater percent reduction in eGFR at 1-year follow-up compared with subjects with high sCD40R (−5.6% ± 0.19% vs. 0.33% ± 0.25%; p = 0.05) (Fig. 1b). We also stratified subjects based on their plasma levels of both sCD40L and sCD40R (Fig. 2). Subjects with high sCD40L/low sCD40R had significantly greater declines in eGFR at 1-year follow-up compared to subjects with low sCD40L/high sCD40R (−7.5% vs. 5.2%; p = 0.02) (Fig. 2).

Multivariable analysis was used to further assess the relationship between sCD40L, sCD40R, and changes in renal function (Table 2). Following adjustment for age, sex, SBP, baseline eGFR, PVD, diabetes, primary renal disease classification, and baseline usage of aspirin or ACEi/ARB medications, high sCD40L was independently predictive of a decline in eGFR percent change at 1-year follow-up (−7.57%, p = 0.014). Low sCD40R was also predictive of a decline in eGFR percent change at 1-year follow-up (−6.39%, p = 0.044). The only other variable in our model predictive of statistically significant changes in eGFR was systolic blood pressure, which predicted a 0.20% decrease in eGFR at 1-year per unit increase in SBP (p = 0.02). As the CKD-EPI equation is also a commonly used indicator of renal function, we tested our model using eGFR calculated with CKD-EPI-creatinine equation, which yielded similar results when compared to MDRD eGFR (S Table 2).

**Table 2:** Independent Predictors of Percent Change in eGFR at 1-Year Follow-up.

Predictors	Effect estimate (% change in eGFR) (95% CI)	Odds Ratio (95% CI)	P-value
High sCD40L	−7.57 (−13.60, −1.54)	1.89 (1.13, 3.10)	0.014
Low sCD40R	−6.39 (−12.59, −0.18)	1.71 (1.01, 2.85)	0.044
Systolic BP	−0.20 (−0.36, −0.04)	1.02 (1.00, 1.03)	0.02

Model was adjusted by age, sex, baseline eGFR, systolic blood pressure (SBP), peripheral vascular disease (PVD), diabetes mellitus, primary renal disease, and baseline ACEi/ARB or aspirin use. Odds ratios were calculated for a negative percent change in eGFR at 1-year follow-up. Only significant predictors are shown.

Abbreviations: *eGFR, estimated glomerular filtration rate; sCD40L, soluble CD40 ligand; sCD40R, soluble CD40 receptor; BP, blood pressure; and CI, confidence interval.*

The impact of circulating CD40 mediators on longitudinal changes (baseline and annually out to 4 years) in renal function was also assessed using a WGEE model (Table 3). Following adjustment for age, sex, baseline eGFR, SBP, PVD, diabetes, primary renal disease, and ACEi/ARB or aspirin use, our model, using the high sCD40L/low sCD40R as the comparator group, identified low sCD40L/high sCD40R as a statistically significant independent predictor of attenuation of renal function decline (3.21 ml/min per 1.73 m^2^ per year; p = 0.016). The other variables in our model that achieved statistical significance were baseline MDRD eGFR, PVD and years (annually out from baseline to 4 years). In particular, baseline eGFR is an important significant predictor in our GEE model as it addresses the dependency of longitudinal repeated measurements of renal function from the same subject. Low sCD40L/high sCD40R was predictive of attenuated renal decline in addition to the expected decline in renal function over time.

**Table 3:** Independent Predictors of Longitudinal Changes in eGFR.

Predictors^*^	Effect estimate (% change in eGFR) (95% CI)	P-value
Low sCD40L & High sCD40R	3.21 (0.59, 5.83)	0.016
Baseline MDRD eGFR	1.01 (0.94, 1.0)	<0.001
Year (out from baseline)	−1.32 (−1.80, −0.85)	<0.001
PVD	1.92 (0.16, 3.67)	0.03

Only statistically significant predictors of longitudinal changes in eGFR (baseline out to 4 years) are included. The model was adjusted for, age, sex, baseline eGFR, systolic blood pressure (SBP), history of peripheral vascular disease (PVD) and diabetes mellitus, primary renal disease, and baseline ACEi/ARB or aspirin use.

*Using High sCD40L & Low sCD40R as the reference group

Abbreviations: MDRD, Modified Diet in Renal Disease study; GFR, estimated glomerular filtration rate; sCD40L, soluble CD40 ligand; sCD40R, soluble CD40 receptor; BP, blood pressure; and CI, confidence interval; and PVD, peripheral vascular disease.

### CD40 and Composite Endpoint

The composite endpoint, defined as the first occurrence of renal replacement therapy (RRT), cardiovascular or renal death, or major adverse cardiac event (either myocardial infarction, congestive heart failure or stroke) (MACE), as well as the secondary component endpoints were examined using Kaplan-Meier estimates and Cox proportional-hazards model adjusting for age, sex, body mass index (BMI), and baseline creatinine and urine protein. No statistically significant differences in the proportion of endpoint events were observed when subjects were grouped by high or low levels of either sCD40L or sCD40R (S Fig. 1). Subjects with high sCD40L were more likely to have an endpoint event, although this trend was not statistically significant.

## Discussion

To the best of our knowledge, this is the largest cohort of all-cause CKD subjects in which plasma levels of both sCD40L and sCD40R were measured. Our results demonstrate that CD40 plasma mediators predict changes in renal function in subjects with CKD.

No differences in the proportion of endpoints events were found when our study population was analyzed by dichotomizing either sCD40L or sCD40R at their medians (S Fig. 1). This finding is corroborated by a recent study conducted by Rusu *et al*.^28^. However, we did observe that subjects with high sCD40L were more likely to experience an adverse event. It should be noted that our study was not sufficiently powered for composite endpoint analysis.

A 2010 prospective observational study conducted by Lajer *et al*. in subjects with type 1 diabetic nephropathy demonstrated that while these subjects had statistically significant higher levels of plasma sCD40L than normo-albuminuric controls, sCD40L levels were not predictive of mortality or decreases in kidney function^29^. In our study, diabetes was the primary cause of CKD in 40 of the 244 subjects (Table 1). Our findings indicate that levels of sCD40L were predictive of decreases in eGFR at 1-year follow-up. It is important to note, however, that our study was not sufficiently powered to specifically determine renal disease progression or mortality in subjects with diabetic nephropathy. Moreover, the baseline eGFR of the Lajer *et al*. study was 76 ± 33 ml/min per 1.73 m^2^ whereas the baseline eGFR in the present study is 29 ± 15 ml/min per 1.73 m^2^. This large difference in baseline eGFR may factor into the discrepancies between our respective studies.

Our results suggest that CD40 signaling is detrimental to kidney function. As discussed previously, CD40-mediated signaling events have been linked to inflammation, atherosclerosis and thrombosis^6, 8, 14–16, 30, 31^. Interestingly, the CD40 receptor has been demonstrated to be expressed on renal parenchymal cells, including the proximal tubules^4, 5^. Prior studies have linked CD40 signaling with proximal tubule inflammation and damage. Up-regulation of proximal tubule-associated CD40 receptor via transforming growth factor beta (TGFβ) mediated pathways led to increased destruction of proximal tubules by CD8 + cytotoxic T cells^32^. Others have shown that activation of proximal tubular-associated CD40 receptor is sufficient to cause a pro-fibrotic and pro-inflammatory response *in vitro* ^7, 33, 34^. Importantly, inhibition of CD40 signaling has been shown to reduce the extent of renal injury in animal models of kidney disease^35, 36^. A recent report by our group suggests that CD40 significantly contributes to the development of renal fibrosis in experimental hypertensive nephropathy^37^.

sCD40R has been shown to directly antagonize CD40 signaling in B cells^10–13^. Moreover, there is evidence to suggest that elevated levels of sCD40R may partially account for end stage renal disease associated immunodeficiency^38^. Given the increasing amount of evidence implicating CD40 in renal injury, sCD40R, an endogenous CD40 antagonist, holds promising potential as a therapeutic target. Importantly, whether sCD40R can directly inhibit CD40-mediated pro-fibrotic and pro-inflammatory signaling in the kidney has not been studied. However, we have previously demonstrated that sCD40R was inversely associated with renal function decline in an atherosclerotic renal artery stenosis cohort^20^. It should be noted that a 2012 study conducted by Esposito *et al*. demonstrated that sCD40R levels and eGFR were inversely correlated (a high level of sCD40R was associated with low eGFR and vice versa) in uremic subjects not on hemodialysis^39^. These opposing findings may be related to the small sample size of the Esposito study (8 subjects). In aggregate, the results from our current study support the hypothesis that sCD40R may be renoprotective. We show that subjects with high sCD40L/low sCD40R had an accelerated decline in renal function compared to subjects with low sCD40L/high sCD40R at both short-term and long-term follow-up. Furthermore, low sCD40R was found to be an independent predictor of renal function decline following adjustment for covariates such as age, sex, systolic blood pressure, baseline eGFR, history of peripheral vascular disease or diabetes, primary renal disease classification, and use of ACEi/ARB medications (Table 2). Our findings clearly necessitate the need for further mechanistic studies to better understand the function of sCD40R in the kidney.

Some limitations of the design of our study need to be considered. As alluded to previously, we were unable to conduct subgroup analyses due to the size of our study. Future studies utilizing a greater sample size could enhance our understanding of the role of CD40 signaling in specific subtypes of CKD classifications. Moreover, we measured baseline plasma levels of sCD40L and sCD40R. Further studies with repeated measurements of plasma sCD40L and sCD40R are required to better elucidate the long-term effects of CD40 signaling mediators in CKD. As a retrospective observational study, we were only able to make associations between circulating CD40 mediators and changes in renal function. Future mechanistic studies are still needed to prove the causative relationship between CD40 and CKD.

A growing body of both basic and clinical research implicates the CD40 pathway as a critical mediator of CKD progression. The present findings demonstrate that baseline levels of sCD40L and sCD40R are predictive of changes in renal function at both short- and long-term follow-up in a CKD cohort. Future studies are needed to determine whether interruption of the CD40 signaling, and specifically, sCD40R, can prevent progression of CKD.

## Electronic supplementary material

Supplementary Materials
