# Supplementary material for: Circulating CD40 and sCD40L Predict Changes in Renal Function in Subjects with Chronic Kidney Disease
Source: Sci Rep. 2017 Aug 11;7:7942. doi: 10.1038/s41598-017-08426-8 (PMC5554219; doi:10.1038/s41598-017-08426-8)
Supplement: Supplementary file 1 — Supplementary Materials [file 41598_2017_8426_MOESM1_ESM.pdf]

Circulating CD40 and sCD40L Predict Changes in Renal Function in Subjects with Chronic Kidney Disease

Jeffrey X. Xie, BS[1], Helen Alderson, MD[2], James Ritchie, MD[2] Philip A. Kalra, MD[2], Yanmei Xie, MA[1], Kaili Ren, Ph.D[1], Hanh Nguyen, MA[1], Tian Chen, Ph.D[1], Pamela Brewster, MA[1], Rajesh Gupta, MD,[1] Lance D. Dworkin, MD[1], Deepak Malhotra, MD, Ph.D.[1], Christopher J. Cooper, MD[1], Jiang Tian, Ph.D. [1] Steven T. Haller*, Ph.D.[1]

[1]University of Toledo College of Medicine and Life Sciences, Toledo, Ohio

[2]Salford Royal Hospital, Salford, United Kingdom

Running Title: CD40 and Chronic Kidney Disease

Key Words: chronic kidney disease, CD40

***Address for Correspondence**:

Steven T. Haller, Ph.D.

University of Toledo College of Medicine and Life Sciences

Department of Medicine

Health Education Building RM205

3000 Arlington Ave.

Toledo, OH 43614

Phone: 419-383-6859

Fax: 419-383-6863

Steven.haller@utoledo.edu

Supplemental Information

S Table 1. Median sCD40L and sCD40R by Type of Primary Renal Disease

**Primary renal disease***	**sCD40L** (p-value = 0.11)***	**sCD40R** (p-value = 0.77)***	**sCD40L**** (p-value = 0.11)***	**sCD40R**** (p-value = 0.77)***
**DM** (n=40)	213.81±188.02	72.63±282.99	5.37±0.79	4.28±1.96
**APKD** (n=16)	144.68±137	97.33±292.68	4.97±0.82	4.58±1.90
**GN/VAS** (n=83)	202.71±18.48	78.15±101.46	5.31±0.84	4.36±1.09
**Pyelonephritis** (n=32)	184.93±203.44	87.68±331.312	5.22±1.01	4.47±1.97
**VAS/HTN** (n=16)	296.46±172.81	125.30±222.41	5.69±0.58	4.83±1.44
**Other** (n=56)	204.27±169.39	84.93±255.83	5.32±0.76	4.44±1.76

*Date are expressed as medians ± interquartile range (IQR)
**sCD40L and sCD40R are measured in the log scale
***Comparisons among renal disease types using nonparametric Kruskal-Wallis rank sum test

Abbreviations: *sCD40L, soluble CD40 ligand; sCD40R, soluble CD40 receptor; DM, diabetic glomerularnephritis; APKD, adult polycystic kidney disease; GN/VAS, glomerularnephritis vasculitis; VAS/HTN, and vascular hypertension*

S Table 2. Independent Predictors of Percent Change in CKD-EPI eGFR at 1-Year Follow-up

Predictors**	Effect estimate (% change in eGFR) (95% CI)	Odds Ratio* (95% CI)	P-value
High sCD40L	-7.95 (-14.43, -1.5)	1.87 (1.12, 3.10)	0.016
Low sCD40R	-6.72 (-13.37, -0.063)	1.70 (1.0, 2.85)	0.048
Systolic BP	-0.20 (-0.37, -0.025)	1.02 (1.00, 1.03)	0.025

*Odds ratio for a negative percent change in CKD-EPI eGFR at 1-year follow-up
**Model was adjusted by age, sex, baseline CKD-EPI eGFR, peripheral vascular disease, systolic blood pressure, diabetes mellitus, baseline angiotensin converting enzyme inhibitor (ACEi) / angiotensin receptor blocker (ARB) or aspirin use, and primary renal disease. Only significant predictors are shown.

Abbreviations*: CI, confidence interval; CKD-EPI, Chronic Kidney Disease Epidemiology Collaboration; eGFR, estimated glomerular filtration rate; BP, blood pressure; sCD40L, soluble CD40 ligand; sCD40R, soluble CD40 receptor*

S Figure 1. Percent incidence and odds ratios of major adverse events for CRISIS participants grouped by sCD40L and sCD40R

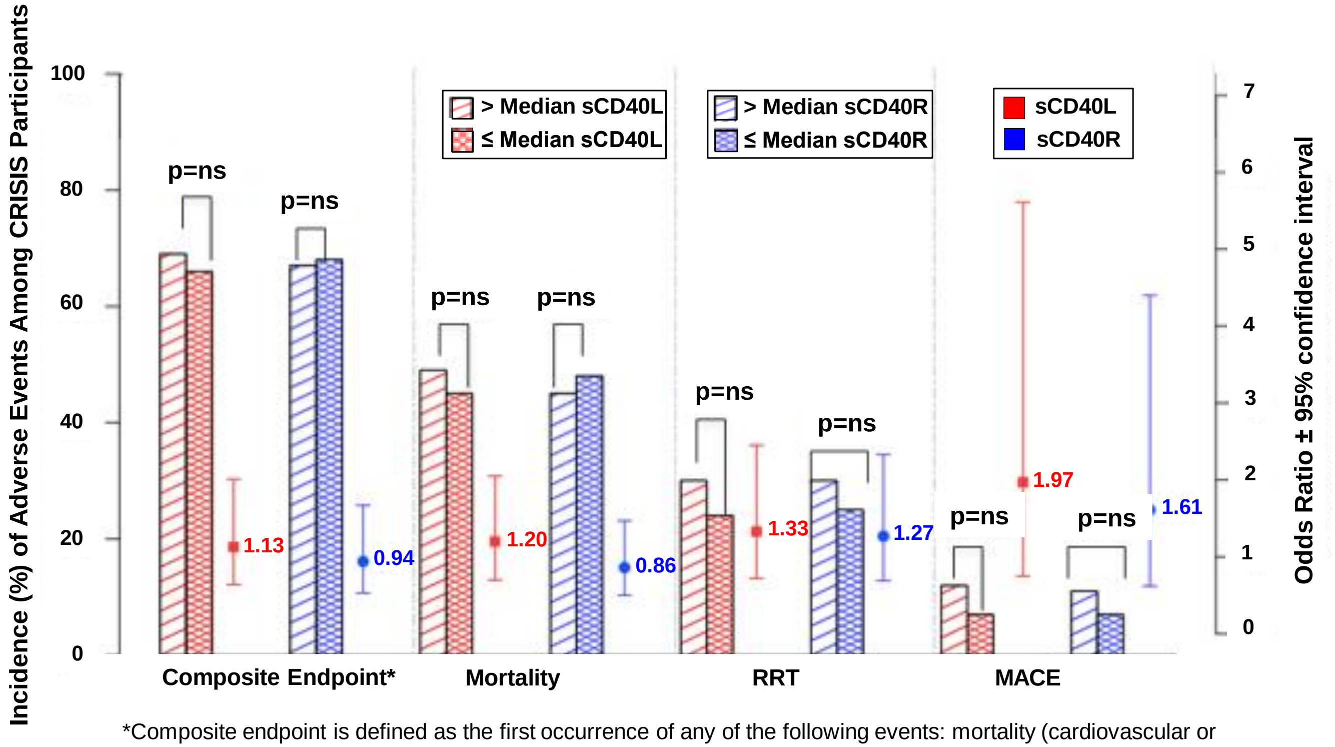

*Composite endpoint is defined as the first occurrence of any of the following events: mortality (cardiovascular or renal death); renal replacement therapy (RRT); and major adverse cardiovascular event (MACE) comprising either myocardial infarction, congestive heart failure or stroke.

Abbreviations: sCD40L, soluble CD40 ligand; and sCD40R, soluble CD40 receptor.
